# A positive feedback loop driven by fibronectin and IL-1β sustains the inflammatory microenvironment in breast cancer

**DOI:** 10.1186/s13058-023-01629-0

**Published:** 2023-03-15

**Authors:** Gurcan Tunali, Hamdullah Yanik, Suleyman Can Ozturk, Secil Demirkol-Canli, Georgios Efthymiou, Kerim Bora Yilmaz, Ellen Van Obberghen-Schilling, Gunes Esendagli

**Affiliations:** 1 Department of Basic Oncology, Hacettepe University Cancer Institute, Ankara, Turkey; 2 Science for Life Laboratory, Department of Immunology, Genetics and Pathology, Uppsala University, Uppsala, Sweden; 3 Research and Application Center for Animal Experiments, Hacettepe University Cancer Institute, Ankara, Turkey; 4 Department of Medical Oncology, Division of Tumor Pathology, Hacettepe University Cancer Institute, Ankara, Turkey; 5 Cancer Research Center of Marseille, INSERM, Marseille, France; 6 Department of General Surgery, Gulhane Faculty of Medicine, University of Health Sciences, Ankara, Turkey; 7 Université Côte d’Azur, CNRS, INSERM, iBV, Nice, France

**Keywords:** Fibronectin, Triple-negative breast cancer, Inflammation, Tumor-associated macrophage, STAT3, NF-κB

## Abstract

Inflammatory alterations of the extracellular matrix shape the tumor microenvironment and promote all stages of carcinogenesis. This study aims to determine the impact of cellular fibronectin on inflammatory facets of tumor-associated macrophages (TAMs) in breast cancer. Cellular fibronectin (FN) harboring the alternatively spliced extra domain A (FN-EDA) was determined to be a matrix component produced by the triple-negative breast cancer (TNBC) cells. High levels of FN-EDA correlated with poor survival in breast cancer patients. The proinflammatory cytokine IL-1β enhanced the expression of cellular fibronectin including FN-EDA. TAMs were frequently observed in the tumor areas rich in FN-EDA. Conditioned media from TNBC cells induced the differentiation of CD206^+^CD163^+^ macrophages and stimulated the STAT3 pathway, ex vivo. In the macrophages, the STAT3 pathway enhanced FN-EDA-induced IL-1β secretion and NF-κB signaling. In conclusion, our data indicate a self-reinforcing mechanism sustained by FN-EDA and IL-1β through NF-κB and STAT3 signaling in TAMs which fosters an inflammatory environment in TNBC.

**Supplementary Information:**

The online version contains supplementary material available at 10.1186/s13058-023-01629-0.

## Introduction

Inflammation is a common facet of cancer that facilitates tumor progression and contributes to disease heterogeneity [1]. Even though breast cancer is well-characterized at cellular and molecular levels, the immune modulation in the tumor microenvironment (TME) remains to be better elucidated [2, 3]. Basal-like and triple-negative breast cancer (TNBC) cells, which are devoid of the estrogen and progesterone hormone receptors (HRs) and do not overexpress the human epidermal growth factor receptor 2 (HER2), is the most aggressive subtype [2, 4]. TNBCs are highly infiltrated by immune cells such as T lymphocytes and TAMs [5]. Under the influence of tumor-derived factors, immune cells contribute to chronic inflammation and promote tumor growth [1]. IL-1β, tumor necrosis factor (TNF)-α and the danger-associated molecular patterns (DAMPs) found in the TME stimulate the nuclear factor-kappa B (NF-κB) pathway [6–8]. The NF-κB and signal transducer and activator of transcription 3 (STAT3) inflammatory pathways are major pathways that drive pro-tumor activities of TAMs [6].

Not only inflammatory mediators, but also the components of extracellular matrix shape the phenotype and function of macrophages [9]. Upon extravasation, monocytes adhere to FN and initiate the monocyte-to-macrophage differentiation program [10, 11]. Under physiological conditions, cellular fibronectin is produced and assembled mainly by fibroblasts. In the TME, cancer-associated fibroblasts (CAFs) are activated to produce high amounts of fibronectin. Fibronectin is a modular protein comprised of different homologous repeats (types I, II, and III) that serve as binding sites for integrins, growth factors, and matrix components, including FN itself [12, 13]. Alternative splicing of the *FN1* gene generates cellular FN variants containing additional type III repeats referred to as extra domains including Extra Domain A and Extra Domain B, that flank the RGD-containing cell binding site of FN. Notably, the Extra Domain A (FN-EDA), that specifically binds to integrins α4β1, α9β1, and α4β7, has been implicated in many biological processes such as cell adhesion, wound healing and tissue remodeling, cell proliferation and differentiation, and inflammation [12]. In addition, FN-EDA serves as a ligand for Toll-like receptor 4 (TLR4), which is upregulated on activated macrophages, and triggers the inflammatory responses through the NF-κB pathway [13, 14].

The involvement of NF-κB and STAT3 pathways in breast cancer has previously been reported [15, 16]. Especially in TNBC, the inflammatory microenvironment promotes the accumulation of TAMs. In addition, high levels of total fibronectin have been detected in the tumor tissue and in the circulation of breast cancer patients [5, 17, 18]. Particularly, the EDA- and EDB-containing isoforms of the cellular fibronectin are upregulated in invasive breast cancers and in TNBC cell lines [19, 20]. Therefore, we set out to explore the impact of FN-EDA on the inflammatory environment in breast cancer. Here, we showed that IL-1β upregulates FN-EDA secretion by TNBC cells. The conditioned media (CM) collected from the TNBC cells induced monocyte-to-macrophage differentiation and stimulated the STAT3 pathway. Moreover, FN-EDA stimulated IL-1β production and NF-κB activation in monocytes. Therefore, our results indicate a link between cancer cells and macrophages through fibronectin and IL-1β which contributes to inflammation in the TME.

## Materials and methods

### Patient samples

Tumor tissue specimens were obtained from the breast cancer patients (*n* = 62, median age 57, min. 41–max. 92) who were treatment naïve and underwent oncological surgery (clinical grade I, *n* = 3; grade II, *n* = 32; grade III, *n* = 16; grade IV, *n* = 1). All tumors were histopathologically categorized as grade 3, and HR expression and HER2 amplification status were evaluated (HR^+^HER2^−^, *n* = 20; HR^+^HER2^+^, *n* = 15; HR^−^HER2^+^, *n* = 10; HR^−^HER2^−^, *n* = 17). Patient data are summarized in Additional file 1: Table S1. Samples from histopathologically normal non-tumor breast tissue were also collected. The freshly obtained tissue samples were stored in a protective solution (RNAlater, QIAGEN, USA) and kept at − 80 °C. The study was approved by our local ethical committee and informed consent was obtained from the participants.

### Cell culture

Breast cancer cell lines MCF-7, T-47D, ZR-75-1, BT-474, SK-BR-3, MDA-MB-468, HCC38, and MDA-MB-231 were cultured in appropriate cell culture media which were supplemented with 10% fetal bovine serum (FBS; Lonza, Switzerland), 1% L-glutamine and 1% penicillin–streptomycin (Biological Industries, Israel), and insulin (0,01 μg/mL; Sigma, USA) for T47D, according to the recommendations of the provider (ATCC, LGC Promochem, USA). THP-1 and U937 myeloid cell lines and the primary peripheral blood CD14^+^ monocytes purified from the healthy volunteers (MACS Human CD14 MicroBead Kit, Miltenyi Biotech, Germany) were cultured in standard RPMI 1640 medium supplemented with 10% FBS and L-glutamine and 1% penicillin–streptomycin. The genetically modified THP-1 cell lines shControl/THP-1, shSTAT3/THP-1, Mock/THP-1, and STAT3DN/THP1 were previously described [21].

To obtain CM from breast cancer lines, cells were incubated in standard RPMI 1640 medium (5 × 10^5^ cells/mL) for 48 h, then the supernatant fraction was collected and applied freshly to the myeloid cells following centrifugation to remove cell debris.

For certain experimental setups, the breast cancer cell lines (5 × 10^5^ cells/mL) were treated with IL-1β (0.4 ng/mL), IL-6 (20 ng/mL), or TNF-α (1.6 ng/mL) (R&D, USA). To inhibit the STAT3-mediated signal transduction, the myeloid cells were pre-incubated for 45 min with previously determined non-toxic doses of a small molecule inhibitor, stattic (5 μM for healthy monocytes and THP-1, 2.5 μM for U937) (Santa Cruz, USA). As a control for stattic, the cells were treated with the solvent DMSO. The incubations with plasma fibronectin (pFN; Sigma), recombinant FN isoforms with and without EDA domain, rFN-EDA^+^ and rFN-EDA^−^, respectively (Additional file 1: Fig. S1) (15 μg/mL) were performed in RPMI 1640 medium containing 1% FBS. The expression and purification of recombinant human fibronectin isoforms containing or lacking the EDA domain, rFN-EDA^+^ and rFN-EDA^−^ proteins, respectively, were previously described [22]. The recombinant protein production was performed using a mammalian expression system; therefore, LPS contamination was avoided [22]. All cell culture experiments were performed in a humidified, 5% CO_2_ incubator at 37 °C.

### Semiquantitative RT-PCR

Total RNA was isolated from the cell lines, or the patient tissue samples (Norgen, Canada) and converted to cDNA (NEB, USA). Quantitative PCRs were performed (SsoAdvanced™ Universal SYBR Green Super Mix, Bio-Rad, USA) with primer oligonucleotides for IL-1β (NM_000576.3), forward 5′-AAGTACCTGAGCTCGCCAGT-3′, reverse 5′-TGGAAGGAGCACTTCATCTGT-3′; fibronectin 1 (NM_212482.4), forward 5′-ACTGCGAGAGTAAACCTGAAGC-3′, reverse 5′-GCGGTTTGCGATGGTACAGCT-3′; STAT3 (NM_139276.3), forward 5′-GATGCGGAGAAGCATCGTGA-3′, reverse 5′-ATCTAGGCAGATGTTGGGCG-3’; FN-EDA (NM_002026.4), 5′-TGCAGTAACCAACATTGATCGC-3’, reverse 5′-TTCAGGTCAGTTGGTGCAGG-3′; β-actin (NM_001101.5), forward 5′-CTGGAACGGTGAAGGTGACA-3′, reverse 5′-AAGGGACTTCCTGTAACAATGCA-3′. Data obtained from target genes were normalized to the housekeeping β-actin gene expression and to those obtained from the control samples. The change in gene expression was calculated with 2e-ΔΔCt or -ΔΔCt formulas, which was previously described [23].

### In silico* analyses*

The METABRIC EGAD00010000211 (Illumina HT-12 v3) normalized microarray data of breast tumors (*n* = 995) has been used for the analyses of gene expression correlations and prognostic assessments [24]. SOFT file for Illumina HT-12 v3 platform (GPL6947) was downloaded from Gene Expression Omnibus (GEO). Probeset IDs for EDA (NM_002026.2) and isoforms lacking the EDA domain (NM_054034.2, NM_054034.2) available in this platform were determined (EDA: ILMN_1778237, isoforms lacking EDA domain: ILMN_1675646, ILMN_2366463) using the SOFT file. M2 macrophage fractions of each sample in METABRIC cohort have been calculated using CIBERSORT Web tool with absolute mode [25]. Samples with deconvolution *p* value less than 0.05 were eliminated to filter out the results with a poor “goodness of fit.”

### Immunofluorescence

Frozen tumor sections were fixed with 4% paraformaldehyde and blocked with 10% bovine serum albumin (BSA). They were incubated with anti‐human phospho-STAT3 Tyr705 (D3A7, 1/400) (Cell Signaling, USA) [21], ‐CD68 (ERP20545, 1/400) [26], ‐fibronectin (FN-EDA; IST-9, 1/400) [27] (Abcam, UK), ‐EpCAM (9C4, 1/400) (BioLegend, USA) [28]. Antibody binding was detected by Alexa488‐ and Alexa555‐conjugated appropriate secondary antibodies (1:1000, Abcam), and the slides were counterstained with 4′,6‐diamidino‐2‐phenylindole (DAPI; 300 nM, Sigma). The slides were mounted with anti-fade solution (Abcam), observed with a 100×/1.45 NA oil objective on an Olympus BX51 fluorescence microscope equipped with a DC5000c CCD camera, and the images acquired were processed using ImageJ (NIH, USA).

### Flow cytometry

Immunophenotyping was performed with the fluorescently labeled monoclonal antibodies mouse anti-human CD11b (*α*_M_ integrin; D12), CD14 (M5E2), CD11c (*α*_*X*_ integrin; S-HCL3), CD40 (5C3), CD80 (L307.4), CD86 (IT2.2) (BD, USA); CD11a (*α*_*L*_ integrin; HI111), CD18 (*β*_2_ integrin; TS1/18), CD29 (*β*_1_ integrin; TS2/16), CD49d (*α*_4_ integrin; 9F10), *β*_7_ integrin (FIB27), CD163 (GHI/61), CD206 (15-2), TLR4 (HTA125) (BioLegend), MD2 (18H10) (Abcam), and fibronectin (P1H11) (R&D, USA). For the intracellular staining, the breast cancer cell lines were brought to suspension, fixed, permeabilized (Cytofix/Cytoperm Buffer, BD), and washed (Perm/Wash Buffer, BD). Following the incubation with specific antibodies, the cells were washed, and analyses were performed on a flow cytometer (FACSAria II, BD). The percentage and median fluorescence intensity (MFI) values were determined according to the autofluorescence or the staining with isotype-matched control antibodies.

### Reactive oxygen species (ROS) and phagocytosis assay

For the assessment of ROS production, freshly isolated monocytes were suspended in phosphate-buffered saline (PBS, 1x) and labeled with 2 nM 2′,7′-Dichlorodihydrofluorescein diacetate (H_2_DCFDA; Anaspec, USA) for 30 min. These cells were incubated for 48 h in control media and CM. For the phagocytosis assay, Texas red-conjugated latex beads (Sigma), which were pre-incubated with human AB serum (at 1:1 ratio, 1 h), were incubated with the monocytes (5 μL/mL) for 4 h in control media and CM. All incubations were carried out at 37 °C in a humidified, 5% CO_2_ incubator. Flow cytometry was used to obtain the H_2_DCFDA MFI values and to determine the percentage of monocytes that engulfed the latex beads.

### Analysis of costimulatory activity

CD4^+^ helper T (Th) cells were purified (> 95%, by fluorescence-activated cell sorting (FACS)) from the peripheral blood mononuclear cell (PBMC) fraction of the healthy donors and labeled with 5 μM carboxyfluorescein succinimidyl ester (CFSE; Invitrogen, USA) as previously described [29]. The Th cells (25 × 10^3^/0.2 mL) were stimulated with an anti-CD3 monoclonal antibody (HIT3a, 25 ng/mL; eBioscience, USA), and co-cultured for 96 h with different proportions of monocytes which were pre-incubated in CM from breast cancer cell lines or in control media for 48 h. The percentage of proliferating Th cells with a dilution in the CFSE fluorescence was assessed by flow cytometry.

### Western blot

Cells were lysed in RIPA buffer supplemented with protease inhibitor cocktail (PIC, 1x), sodium fluoride (NaF, 200 mM), phenylmethylsulfonyl fluoride (PMSF, 1 mM), ethylenediaminetetraacetic acid (EDTA, 5 mM) and calyculin A (50 nM, Cell Signaling, USA). For the preparation of protein samples from CM, PIC (1x) and EDTA (5 mM) were added into the cell culture supernatant fraction. Following the quantitation with colorimetric bicinchoninic acid (BCA) assay, total protein (50–120 μg) was subjected to SDS-PAGE on 10% gel and transferred onto a polyvinylidene difluoride (PVDF) membrane. The membrane was blocked with 5% skimmed milk powder in PBS-T buffer and incubated overnight at 4 °C with primary antibodies reactive with human phospho-STAT3 Tyr705 (pSTAT3; D3A7, 1/1500), total STAT3 (tSTAT3; 79D7, 1/1500), and β-actin (D6A8, 1/5000) (Cell Signaling), fibronectin (10/Fibronectin, 1/1000) (BD), FN-EDA (IST-9, 1/1000) (Abcam, UK). Following the incubation with HRP-conjugated secondary antibodies (goat anti-rabbit IgG, 1/3000; horse anti-mouse IgG, 1/1500) (Cell Signaling) (1.5 h, at room temperature), and then, with ECL substrate solution, protein bands were visualized and densitometric analyses were performed (Kodak Gel Logic 1500, Carestream, USA). Otherwise stated, all materials were purchased from Thermo Scientific, USA.

### Chemotaxis assay

Monocytes, which were treated with the CM from breast cancer cells or with the control media, were washed thoroughly and resuspended in RPMI 1640 containing 1% FBS. These monocytes (10^5^ cells/100uL) were added into Boyden chambers (8 μm pore size) (Corning, USA) which were placed into the wells containing standard RPMI 1640 medium (10% FBS). Following an 8h incubation, the upper side of the membrane was swabbed to remove the remaining cells, the membrane was mounted and stained with Giemsa (Merck, Germany), and the cells that migrated to the lower membrane surface were counted under a light microscope.

### Polarization assay

Chamber slides (BD) coated with fibronectin (Sigma) or Matrigel (Corning) were used. Monocytes were seeded and then incubated for 16 h with the CM obtained from the breast cancer cell lines. Thereafter, slides were fixed in 4% paraformaldehyde for 30 min, treated with 0.1% Triton X-100 (Sigma), and blocked with 10% BSA (Sigma). The cells were stained with Phalloidin-iFluor 488 (1:1000, Abcam) and DAPI (300 nM, Sigma). The morphological polarization of cells was analyzed by fluorescence microscopy and the images were processed (ImageJ, NIH, USA). The polarization was assessed according to the images of actin cytoskeleton stained with phalloidin. A polarization index was calculated on ImageJ software where an operation image filter was applied (Directionality; Fourier Components Analysis). Distribution, amount, and goodness were obtained after filtering and the cell polarity index was calculated as arbitrary units with the formula: Polarity index = Amount × Goodness/[(180 − distribution)/180].

### Enzyme-linked immunosorbent assay (ELISA)

Supernatants were collected from the breast cancer cell lines (5 × 10^5^ cells/mL, 48 h) and the monocytes (5 × 10^5^ cells/mL, 24 h) incubated in CM from the breast cancer cell lines with or without recombinant human fibronectin isoforms rFN-EDA^+^, rFN-EDA^−^, or pFN (15 µg/mL, 16 h). ELISA kits for human fibronectin (Abcam) and IL-1β (BioLegend) were used according to the manufacturer’s instructions.

### Reporter assay

The monocytes were incubated in CM from the breast cancer cell lines with or without the isoforms of fibronectin (15 µg/mL). Then, the supernatants were collected and applied (20 µL) onto HEK-Blue™ cells (5 × 10^4^ cells/180 μL; Null2, InvivoGen, USA), which carry a secreted embryonic alkaline phosphatase (SEAP) reporter gene under the control of NF-κB, for 16 h in HEK-Blue™ Detection medium (InvivoGen). SEAP activity was evaluated at 650 nm (SpectraMax Plus, Molecular Devices, USA).

### Statistical analyses

Statistical tests and graphical outputs were performed on GraphPad Prism 6 Software (Prism, San Diego, USA). Cutoffs for the determination of groups in Kaplan–Meier plots were chosen as the expression value at which the lowest log-rank *p* value is obtained within the interquartile range as described previously [30]. Student’s paired or unpaired t tests or ANOVA with post hoc analyses were used where appropriate. A “*p*” value lower than 0.05 was considered as an indicator of statistical significance. Otherwise noted, all data are given as the mean ± SEM.

## Results

### FN-EDA expression is associated with triple-negative breast cancer and enhanced by IL-1β

**Fig. 1:**
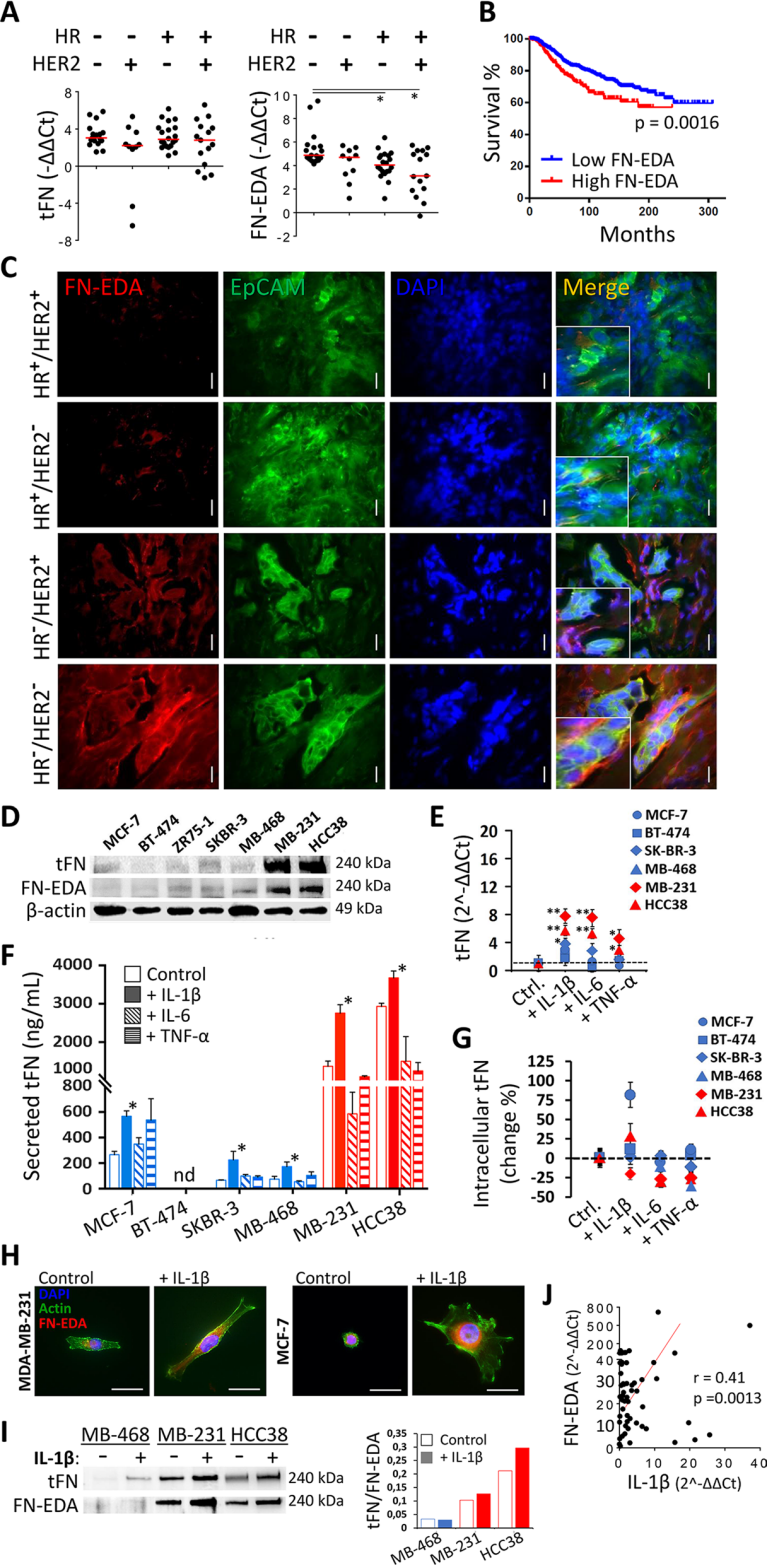
Expression of total fibronectin (tFN) and the alternatively spliced variant of cellular fibronectin harboring the extra domain A (FN-EDA) in breast cancer. **A** Gene expression of tFN and FN-EDA was evaluated in freshly obtained treatment-naïve breast tumor samples (*n* = 62) by quantitative RT-PCR. Quantitation of the target gene expression was first normalized to the housekeeping β-actin gene expression for each sample. Then, the Ct values from the tumor-adjacent histopathologically normal breast tissue were used for additional normalization of the data. Results were distributed according to hormone receptor (HR) and HER2 status. **B** Kaplan–Meier plot for the estimation of survival in breast cancer patients (*n* = 995, from METABRIC database) with low and high FN-EDA expression. **C** Immunofluorescence analysis of FN-EDA in the breast tumor specimens. Representative images of epithelial cellular adhesion molecule (EpCAM) and FN-EDA are demonstrated for each breast cancer subtype according to HR and HER2 expression. Large magnifications of the merged images are given as indents. The micrographs from one patient analyzed out of eight are shown for each subtype. (Scale bar, 10 µm). **D** Total FN and FN-EDA protein levels were determined in breast cancer cell lines by Western blot. **E** Change in tFN mRNA expression upon treatment with recombinant IL-1β, IL-6, or TNF-α in breast cancer cell lines was analyzed by RT-PCR. Quantitation of the target gene expression was first normalized to the housekeeping β-actin gene expression. Then, the Ct values from the control (untreated) cells were used for additional normalization. The dashed line crossing at 2.^−ΔΔCt^ = 1 represents an equal expression level with the untreated control cells. The change in gene expression was calculated with 2e-ΔΔCt or -ΔΔCt formulas, which was previously described [23]. The data from high-level fibronectin-expressing TNBC cancer cell lines MDA-MB-231 and HCC38 are shown in red. **F** The total FN secretion was assessed by ELISA and **G** the change in cell-associated (both intracellular and surface-bound) tFN levels were assessed by flow cytometry in the breast cancer cell lines stimulated with IL-1β, IL-6, or TNF-α for 24 h. **H** The impact of IL-1β on FN-EDA expression and cell morphology was assessed by immunofluorescence (scale bar, 40 µm). Representative micrographs are shown for MCF-7 and MDA-MB-231 cells. **I** Total FN and FN-EDA levels secreted into the culture media of breast cancer cell lines upon IL-1β treatment. Western blot bands were quantified, and the proportion of tFN to FN-EDA was shown in the bar graph on the right. **J** A correlation analysis between IL-1β and FN-EDA gene expression levels from breast tumor samples. The cell line data are from at least three independent experiments (Mean ± SEM, Student’s t test; *, *P* < 0.05; **, *P* < 0.01)

The triple-negative breast cancers (TNBCs) and the basal-like breast cancers (BLBCs) are characterized by mesenchymal features [2, 31]. Firstly, we sought to determine the expression of the *FN1* gene in patient-derived tumor specimens. Compared to the non-tumor breast tissue, the expression of all transcriptional variants of the *FN1* gene (total fibronectin) was higher in the tumors (Fig. 1A). When the data were distributed according to the HR and HER2 status of the patients, no difference was found in total fibronectin levels; however, FN-EDA-containing variants were significantly upregulated in the triple-negative tumors compared to the HR^+^ samples (Fig. 1A). Analysis of an international cohort database of invasive breast cancers revealed that higher expression of FN-EDA variants significantly associated with decreased overall survival (Fig. 1B). Accordingly, a high-level expression of *FN1* gene (total cellular FN) was also associated with poor prognosis (Additional file 1: Fig. S2).

FN-EDA positivity was observed in HR^−^ tumors (both HER2^+^/HER2^−^), as determined by immunofluorescence staining of tumor tissue (Fig. 1C). In the triple-negative tumors, FN-EDA staining was the most prominent in the EpCAM-expressing tumor cells (Fig. 1C). Additional micrographs from different patients are shown in Additional file 1: Fig. S3. Expectedly, FN-EDA staining was also observed in the EpCAM-negative compartment of the TME where stromal cells, such as CAF, reside (Fig. 1C).

Fibronectin was also barely detectable by western analysis in lysates of HR-positive cell lines (MCF-7, ZR75-1, and SKBR-3) whereas both total fibronectin and FN-EDA protein levels were elevated in the TNBC cell lines MDA-MB-231 and HCC38. Interestingly, the triple-negative MDA-MB-468 line displayed lower FN expression (Fig. 1D). In terms of molecular classification, MDA-MB-231 and HCC38 are categorized as basal-like B (more mesenchymal morphology), whereas the MDA-MB-468 cell line with low FN expression is basal-like A (more epithelial morphology) [32]. When the breast cancer cell lines were stimulated with proinflammatory cytokines both mRNA (Fig. 1E) and secretion (Fig. 1F) of total fibronectin were enhanced, especially with IL-1β. IL-6 and TNF-α were able to induce the fibronectin gene expression, but these cytokines failed to significantly increase the amount of secreted protein (Fig. 1E and F). Notably, the presence of IL-1β increased the production of fibronectin, not only in triple-negative cell lines but also in the HR^+^ lines. In terms of fibronectin expression, MCF-7 was the most responsive HR^+^ cell line to IL-1β (Fig. 1E–G). Intriguingly, the cell-associated levels of fibronectin (both intracellular and surface-bound FN assessed by flow cytometry), were decreased in certain cell lines and especially upon treatment with IL-6 or TNF-α. These cytokines tended to decrease the amount of secreted fibronectin from MDA-MB-231 and HCC38 cells as well (Fig. 1F, G). Morphologically, IL-1β stimulated the spreading of MDA-MB-231 and MCF-7 cells which were sparsely seeded and increased the membrane projections (Fig. 1H). The increase in the EDA-containing FN isoforms was visible in the perinuclear areas of the IL-1β-treated cells (Fig. 1H). Moreover, IL-1β enhanced the secretion of both total fibronectin and FN-EDA as determined in the supernatant fractions (Fig. 1I). A modest correlation was also determined between IL-1β and FN-EDA gene expression in patient tumor specimens (Fig. 1J).

Collectively, cellular fibronectin and its EDA-containing isoforms are positively influenced by the proinflammatory cytokine IL-1β and preferentially expressed in TNBCs.

### Macrophage differentiation and activation are promoted by FN-EDA-expressing triple-negative breast cancer cells

**Fig. 2:**
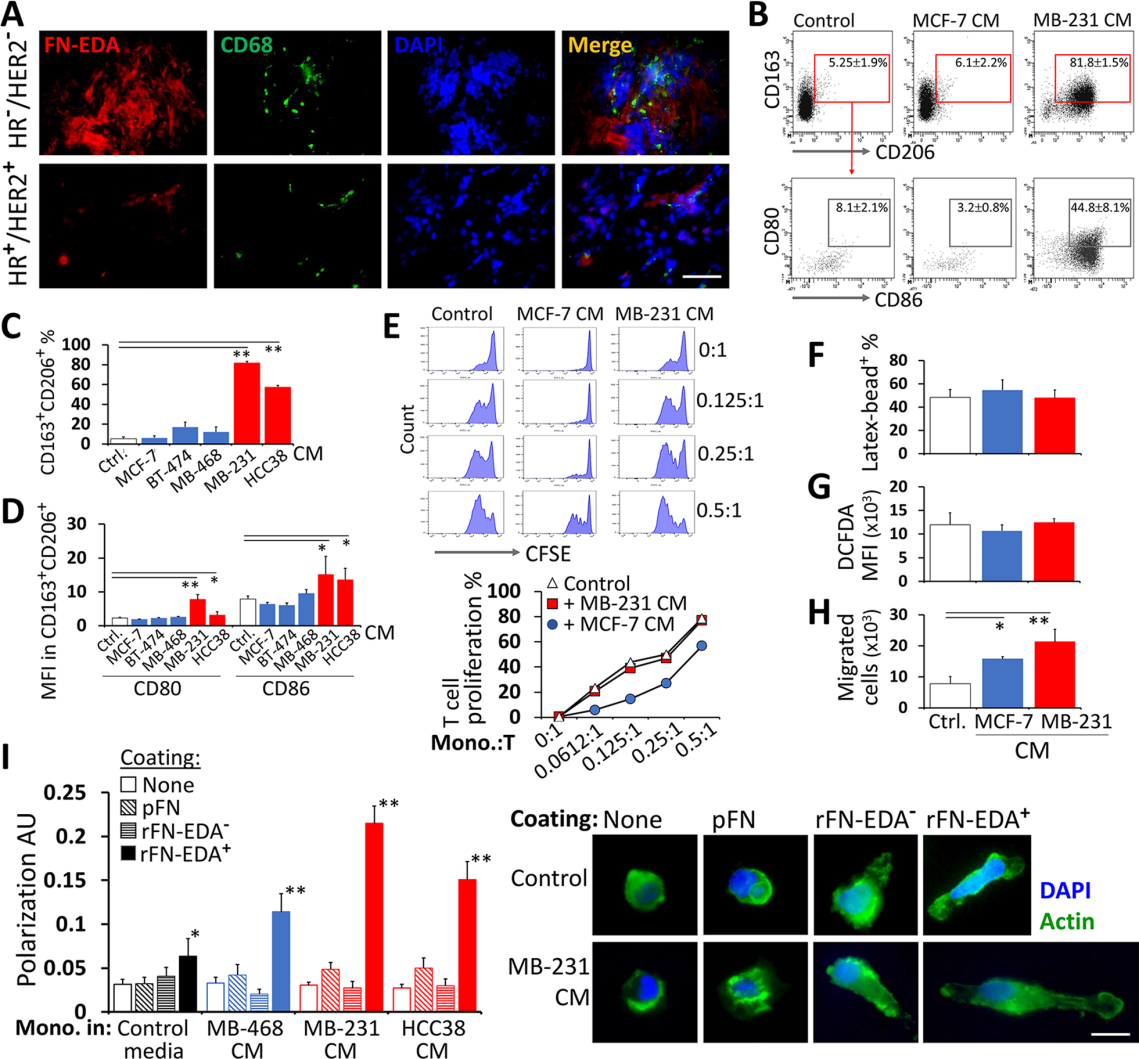
The impact of FN-EDA on macrophage functions in breast cancer. **A** FN-EDA and CD68 immunofluorescence staining in HR- and HER2-positive breast cancer and in triple-negative breast cancer (TNBC) tissue (scale bar, 20 µm). **B**–**D** Freshly isolated monocytes were incubated for 48 h with CM from HR^+^ breast cancer cell lines and from TNBC cell lines. The data from high-level fibronectin-expressing TNBC cancer cell lines MDA-MB-231 and HCC38 are shown in red. Expression of CD163, CD206, CD80, and CD86 macrophage differentiation and activation markers was assessed by flow cytometry. **B** Representative flow cytometry dot plots show the gating strategy for the monocytes treated with CM from HR^+^ MCF-7 and TNBC MDA-MB-231 cells. **C** Percentage of CD163^+^CD206^+^ macrophages and **D** median fluorescence intensity (MFI) value of CD80 and CD86 on CD163^+^CD206^+^ cells. **E** Increasing amounts of monocytes treated with MCF-7 or MDA-MB-231 CM were co-cultured with T cells. T cell proliferation was assessed by flow cytometric CFSE dilution assay. **F** Phagocytosis, **G** production of reactive oxygen species, and **H** migration capacity of monocytes following the treatment with MCF-7 or MDA-MB-231 CM. **I** Polarization of monocytes on the surfaces coated with the plasma fibronectin (pFN), the recombinant FN lacking the EDA domain (rFN-EDA^−^) and the recombinant FN with the EDA domain (rFN-EDA^+^). The cells were pre-treated with CM from the TNBC cell lines. MDA-MB-468 cell line represents a TNBC cell line with low-level fibronectin expression (A.U, arbitrary units). Fluorescence microscopy images of actin staining for monocyte polarization analysis on different surfaces are presented on the right-hand side (scale bar, 40 µm). Each experiment was repeated at least three times with the monocytes from different donors. (Mean ± SEM, Student’s t test; *, *P* < 0.05; **, *P* < 0.01)

TNBCs are frequently infiltrated by TAMs [33]. IL-1β is highly produced by the macrophages, and FN-EDA contributes to macrophage activation [14, 34]. Therefore, we hypothesized that factors secreted from the TNBC cells, including fibronectin, can mediate monocyte-to-macrophage differentiation. Indeed, CD68^+^ TAMs were localized in FN-EDA-rich areas of human breast tumors, as shown in Fig. 2A. Incubation of peripheral blood monocytes in CM directly collected from the TNBC cell lines led to significant upregulation of macrophage markers CD163 and CD206 (Fig. 2B and C). Moreover, these CD163^+^CD206^+^ cells expressed higher levels of CD80 and CD86 activation markers (Fig. 2B and D). They were also capable of providing co-stimulatory signals for T cell proliferation (Fig. 2E). In terms of phagocytosis and ROS production, no difference was observed between the cells exposed to CM from MCF-7 or MDA-MB-231 cells (Fig. 2F and G). In contrast, treatment with the CM from either cell line enhanced the chemotactic activity of the monocytes (Fig. 2H). Collectively, these results indicate that the CM from FN-EDA-expressing tumor cell lines enhanced the functional maturation and motility of monocytes.

The expression of potential receptors for fibronectin, molecules of TLR4 receptor complex and α4, αL, αM, αX, β1, β2, β7 integrins, was not changed following treatment with the CM. CM from MDA-MB-231 cells did increase the expression of CD14 and β2 integrin (Additional file 1: Figs. S4 and S5). To better determine the impact of FN-EDA on activation-related motility of macrophages, the monocytes incubated in the CM from TNBC cells were placed onto surfaces coated with different isoforms of fibronectin. The impact of FN-EDA on the morphological polarization of macrophages was striking (Fig. 2I). Moreover, the cells treated with CM from MDA-MB-231 and HCC38 had the highest polarization scores and displayed a spindle-like shape which is a characteristic of activated macrophages (Fig. 2I).

All in all, the factors secreted from the TNBC cells tend to induce macrophage differentiation. These monocyte-derived macrophages display activation-related features and become morphologically polarized when exposed to FN-EDA.

### Triple-negative breast cancer-derived factors and FN-EDA contribute to inflammatory modulation in macrophages

**Fig. 3:**
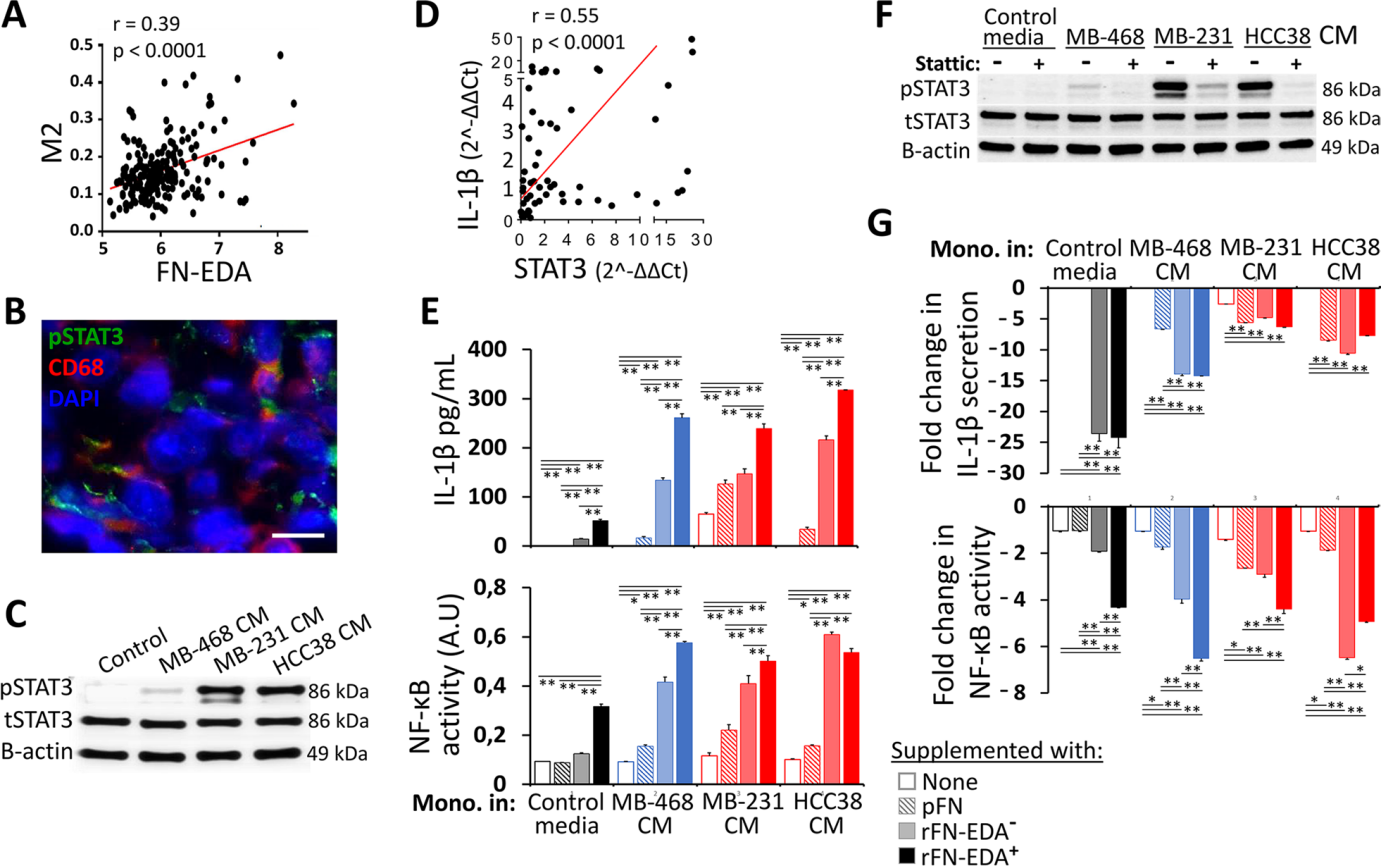
Association of FN-EDA with IL-1β and STAT3 inflammatory pathways. **A** Correlation of macrophage M2 subtype with FN-EDA expression in breast tumors assessed by using METABRIC data. **B** Phospho-STAT3 expression was determined in CD68^+^ macrophages in TNBC tumor specimens. Representative immunofluorescence staining from a patient is shown. **C** STAT3 phosphorylation (pSTAT3) levels were assessed by Western blot in the monocytes treated with CM from TNBC cell lines. MDA-MB-468 cell line represents the Basal-like A subtype of TNBC. **D** Correlation analysis of IL-1β and STAT3 gene expression in freshly obtained breast cancer tissues. **E** IL-1β secretion levels (upper panel) and NF-κB activity (lower panel) in the monocytes incubated with CM from TNBC cell lines in the presence of plasma fibronectin (pFN), recombinant FN lacking EDA domain (rFN-EDA^−^) and recombinant FN with EDA domain (rFN-EDA^+^) (A.U, arbitrary units). **F** Inhibition of TNBC CM-induced activation of the STAT3 pathway through stattic treatment was studied by Western blot. The control cells were treated with DMSO, the solvent for stattic. **G** Decrease in IL-1β secretion (upper panel) and NF-κB activity (lower panel) in the monocytes pre-treated with stattic. The fold change was calculated in comparison to the control monocytes incubated with CM from TNBC cell lines in the presence of the plasma fibronectin (pFN), the recombinant FN lacking the EDA domain (rFN-EDA^−^) and the recombinant FN with the EDA domain (rFN-EDA.^+^). (A.U, arbitrary units). Each experiment was repeated at least three times with monocytes from different donors. (Mean ± SEM, Student’s *t* test; *, *P* < 0.05; **, *P* < 0.01). (Black bars, monocytes in the control media; blue bars, CM from low-level fibronectin-expressing cell lines; red bars, CM from high-level fibronectin-expressing cell lines)

CD163^+^CD206^+^ macrophages have been classified as belonging to the M2-subtype, which favors tumorigenesis and cancer progression [35]. Although, the macrophages generated here are not expected to completely represent TAMs as in vitro conditions used cannot fully recapitulate the TME. Nevertheless, activation of STAT3 pathway is a signature of the M2 phenotype and TAM differentiation [36, 37]. By using the international cohort database, expression of M2-related genes could be associated with FN-EDA mRNA levels in invasive breast cancers (Fig. 3A). As expected, in TNBC tumor sections, TAMs were localized in the FN-EDA-rich matrix and pSTAT3 (Tyr705) was detected in the CD68^+^ macrophages (Fig. 3B). Accordingly, CM from MDA-MB-231 and HCC38 cell lines induced strong activation of the STAT3 pathway in monocytes (Fig. 3C). The ability of culture medium conditioned by TNBC cell lines to increase the pSTAT3 levels in monocytes was also demonstrated in the monocytic cell lines THP-1 and U937. Intriguingly, this effect was not observed using the CM obtained from a panel of HR- or HER2-positive breast cancer cell lines (Additional file 1: Fig. S6). Of note, the expression of total STAT3 was not significantly changed upon the addition of tumor cell CM (Fig. 3C and Additional file 1: Fig. S6). In patient-derived primary tumor tissues, the expression of STAT3 and IL-1β displayed a positive correlation (Fig. 3D). Furthermore, a transient upregulation of IL-1β mRNA levels was identified in the THP-1 cells treated with CM from TNBC cells (Additional file 1: Fig. S7).

Next, we tested whether the proinflammatory activity of monocytes pre-treated with the CM from TNBC cells for 48 h was enhanced in response to different isoforms of FN. Both IL-1β secretion and NF-κB activity were increased in monocytes cultured in control media containing FN-EDA (Fig. 3E). In general, pre-treatment with the CM augmented the inflammatory responsiveness of the monocytes to all FN isoforms used (Fig. 3E). The highest levels of IL-1β and NF-κB were detected using FN-EDA^+^; nevertheless, these cells also moderately responded to the recombinant FN lacking the EDA domain (FN-EDA^−^ and FN-EDB^−^) and minimally to plasma FN (pFN) (Fig. 3E).

When the STAT3 pathway was hindered with a small molecule inhibitor, stattic (Fig. 3F), a significant reduction was observed in the proinflammatory parameters of the monocytes. The reduction in NF-κB activity upon STAT3 inhibition is in line with the impact of recombinant FN-EDA stimulation (Fig. 3G). The negative impact of stattic on NF-κB activity was more notable than on IL-1β production in the monocytes (Fig. 3E and G). In comparison to STAT3 competent cells, the impact of CM on the expression of IL-1β gene was decreased in THP-1 cells in which the STAT3 pathway was hindered through genetic modification with shSTAT3 or double-negative (DN) STAT3 (Additional file 1: Fig. S8).

## Discussion

**Fig. 4:**
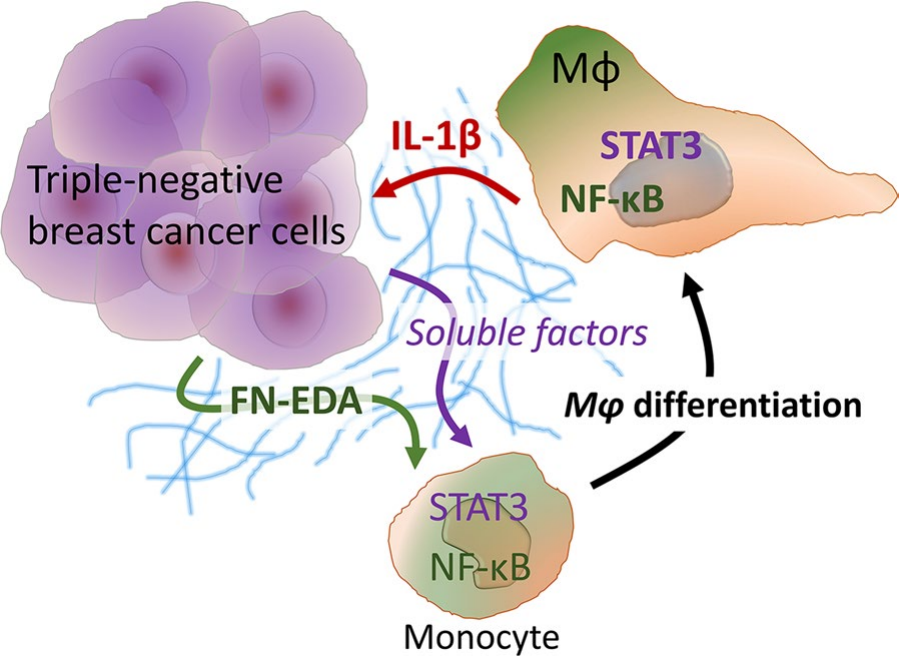
Schematic demonstration of the major findings. Together with the other factors secreted by the triple-negative breast cancer cells, FN-EDA-containing isoforms promote macrophage differentiation through NF-κB and STAT3 pathways and increase IL-1β production. In turn, IL-1β upregulates the expression of FN-EDA by the breast cancer cells to create a self-reinforcing proinflammatory tumor-promoting milieu

Chronic inflammation fuels the progression of cancer where the immune profile in the TME is highly associated with stromal composition and integrity [6]. Accordingly, immune infiltrate is frequently observed in the peritumoral regions and in areas populated by non-malignant cells (e.g., CAF) and extracellular matrix [38–40]. Fibronectin not only paves the way for leukocyte migration into the tumor mass but also its isoform FN-EDA serves as an activation signal for monocytes and macrophages [13, 14, 41]. Our results demonstrate a novel contribution of tumor cell-derived FN-EDA to the development of the inflammatory milieu in breast cancer. The abundance of FN-EDA was a common feature of TNBC cells and directly associated with proinflammatory macrophage activation through IL-1β and STAT3 pathways. The major findings of our study are schematized in Fig. 4.

The extracellular matrix contributes to epithelial–mesenchymal transition and cell migration [42, 43]. Fibroblasts and myoepithelial cells are responsible for producing the majority of extracellular matrix components such as fibronectin [44, 45]. Accordingly, fibronectin production by TNBC cells has been attributed to their myoepithelial origin [44]. Transition from ductal carcinoma in-situ to invasive cancer is correlated with the expression of fibronectin. Increased levels of tumor-derived fibronectin are also inversely correlated with survival [19, 45, 46]. Accordingly, in our study FN-EDA-containing isoforms of fibronectin were abundant in TNBC and associated with poor prognosis. The production by cancer cells of a mesenchymal marker, namely FN-EDA, is clearly in line with the display of mesenchymal features that represent a hallmark of the Basal-B subtype of TNBC [32]. Moreover, it was not surprising that the Basal-A subtype of TNBC cells characterized by an epithelial (luminal) morphology and gene signature (i.e., MDA-MB-468 cell line), did not express total or FN-EDA-containing fibronectin [47]. Induction of mesenchymal-like features and FN-EDA production were simultaneously observed upon IL-β treatment in most of the breast cancer cells independent of their ER positivity. Therefore, our results underline the impact of local inflammation, which is through the induction of FN production as a marker for the epithelial-to-mesenchymal transition, on disease progression in breast cancer.

Induction of mesenchymal features and fibronectin expression upon exposure to proinflammatory cytokines can be cell type- or context-dependent [48–51]. In terms of fibronectin production, the breast cancer cells used in our study were more responsive to IL-1β than to TNF-α or IL-6. As a drawback of our study, we did not analyze the competence of breast cancer epithelial cells to assembly and organize FN produced. The positive influence of IL-1β on fibronectin production is also observed in fibroblasts and endothelial cells [50, 51]. In contrast, IL-1β treatment of lung cancer cells, which induces a mesenchymal-like morphology, does not upregulate fibronectin [52]. TNF-α and IL-6 proinflammatory cytokines can enhance mRNA levels in several cell lines. However, increases in the expression of fibronectin gene do not always lead to increased production and secretion of the protein due to the complexity of posttranslational and secretory mechanisms [53]. At the protein level, both cell-associated and secreted FN were increased in IL-1β-induced MCF-7 cells. Alternatively, the basal-like cell lines had no change or decreased intracellular FN while having a significant increase in secreted FN upon IL-1β treatment. It might be speculated that IL-1β can enhance the secretion of FN that is already produced at high levels by the basal-like cells and this purging of FN into the extracellular milieu might have resulted in a rapid decrease in the stored intracellular FN. On the other hand, upon acquisition of an IL-1β-induced mesenchymal-like phenotype, both the production and the secretion of FN protein may be steadily and positively regulated in MCF-7 cells. Nevertheless, further research is required to better define the impact of IL-1β on FN production and secretion machinery.

Macrophage infiltration has been correlated with the firmness of the stromal areas determined by the organization and composition of the extracellular matrix [54]. Increased levels of TAMs are frequently observed in HR-negative and high histological-grade breast tumors [5]. Moreover, a fibronectin-rich microenvironment has been associated with poor prognosis in breast cancer [45]. In our study, high levels of FN-EDA, which was a common feature of TNBC, were correlated with poor survival and TAMs were frequently detected in the FN-EDA-rich areas of the tumors. Adhesion to fibronectin not only mediates leukocyte migration and localization but also contributes to monocyte-to-macrophage differentiation and activation [10, 41]. FN-EDA binds to integrin heterodimers and to TLR4, CD14, and MD2 receptor complex proteins which are widely expressed in myeloid cells [13, 14]. Proteolytic cleavage of FN, and release of EDA domain-containing fragments, is sustained by the inflammatory infiltrate, and the proteases are widely found in the dysregulated milieu of the tumor [13, 55]. Therefore, in addition to the tumor stroma and inflammatory factors, the FN-derived molecules might play a significant role in the maintenance of an inflammatory milieu through the tumor-infiltrating macrophages. Accordingly, the secreted factors, which include high levels of FN-EDA from TNBC cells induced the differentiation of monocytes into CD206^+^CD163^+^pSTAT3^+^ macrophages with higher migratory capacity, IL-1β production, and NF-κB activity. These findings can be directly associated with the functional and phenotypic profile of TAMs which links the complexity of the TME with the aggressiveness of TNBC. TAMs can simultaneously display the assets of both classically activated proinflammatory M1 and alternatively activated anti-inflammatory M2 subtypes of macrophages [56]. In addition to the robust upregulation of M2-related markers such as CD163, CD206, and STAT3 pathway, these TNBC-induced macrophages also expressed M1-associated markers such as IL-1β, class II MHC molecules, and costimulatory molecules, CD80 and CD86, and did not interfere with T cell proliferation, in vitro. In a previous study from our group, BLBC cells were identified with capacities favoring the inflammatory reactions that may eventually maintain the T cell responses and lead to the generation of regulatory T (Treg) subset which interferes with anti-tumor immunity [29]. Therefore, the interaction between immune cells and tumor cells facilitates the acquisition of an inhibitory character that may contribute to immune evasion [29, 57]. As a drawback of our study, we did not analyze the functional characters of T cells stimulated with the MB-231 CM-treated monocytes.

The STAT3 is a prominent transcription factor for the differentiation and regulatory functions of M2 macrophages and TAMs [36, 58]. It has a well-acknowledged role in tumorigenesis, malignant progression, and immune evasion of TNBC. Furthermore, a reciprocal interaction between the STAT3 pathway and IL-1 signaling has been reported in different biological contexts [59, 60]. Our results delineate the interplay between these pathways as a critical factor in maintaining the inflammatory microenvironment in TNBC. Even though we did not analyze the TNBC-derived factors leading to induction of the STAT3 pathway, TNBC cells are known to secrete many factors such as IL-6, IL-10, VEGF, and TGF-α which can potently stimulate the phosphorylation of STAT3 [15, 42, 59, 61]. STAT3 activity was evident in the macrophages infiltrating the FN-EDA-rich areas of breast tumors. Therefore, in a microenvironment directly provisioned by the TNBC cells, macrophages are exposed to (i) a fibronectin-enriched extracellular matrix, (ii) factors that modulate their differentiation program through STAT3 pathway, and (iii) FN-EDA-mediated induction of IL-1 and NF-κB signaling. Therefore, our findings support the self-sufficiency of TNBC cells in terms of subverting the macrophage-mediated responses and creating an inflammatory milieu that favors cancer progression.

In conclusion, the interaction between TNBC cells and TAMs exploits the positive feedback between FN-EDA and IL-1β, fosters STAT3 and NF-κB pathways, and therefore, may serve as a potential target for damaging malignant progression.

## Supplementary Information

**Additional file 1**. Supplementary Data.

## Data Availability

Not applicable. All datasets used are publicly available and mentioned in the manuscript.
