# Supplementary material for: A positive feedback loop driven by fibronectin and IL-1β sustains the inflammatory microenvironment in breast cancer
Source: Breast Cancer Res. 2023 Mar 15;25:27. doi: 10.1186/s13058-023-01629-0 (PMC10015813; doi:10.1186/s13058-023-01629-0)
Supplement: Supplementary file 1 — Additional file 1. Supplementary Data. [file 13058_2023_1629_MOESM1_ESM.pdf]

Supplementary Material

Suppl. Table 1. Patient data.

**Total number** (n)	62
**Age** median (min-max)	57 (41-92)
**Clinical stage**	
I	3
II	32
III	16
IV	1
**Receptor Status**	
HR^+HER2^-	20
HR^+HER2^+	15
HR^-HER2^+	10
HR^-HER2^-	17

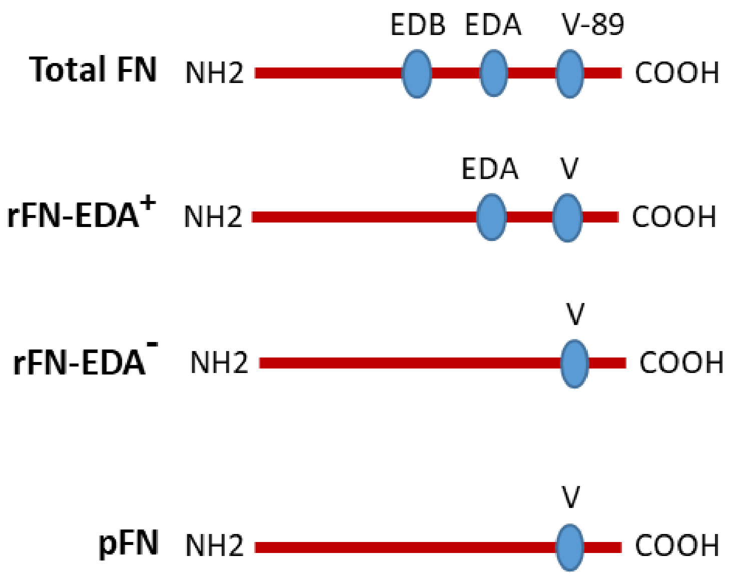

Suppl. Fig. 1. Graphic presentations of the fibronectin (FN) isoforms. The plasma fibronectin (pFN), the recombinant FN lacking the EDA domain ($rFN\text{-}EDA^-$) and the recombinant FN with the EDA domain ($rFN\text{-}EDA^+$) are shown in comparison to the total FN protein.

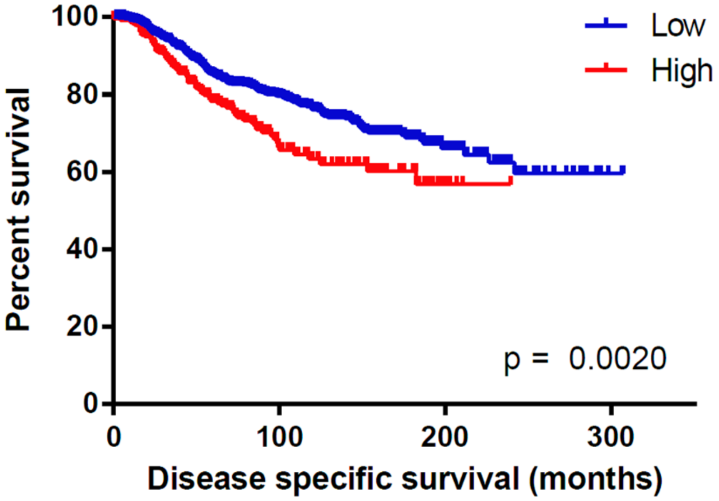

Suppl. Fig. 2 Kaplan-Meier plot for the estimation of survival in breast cancer patients (n=995, from METABRIC database) with low and high total cellular FN expression.

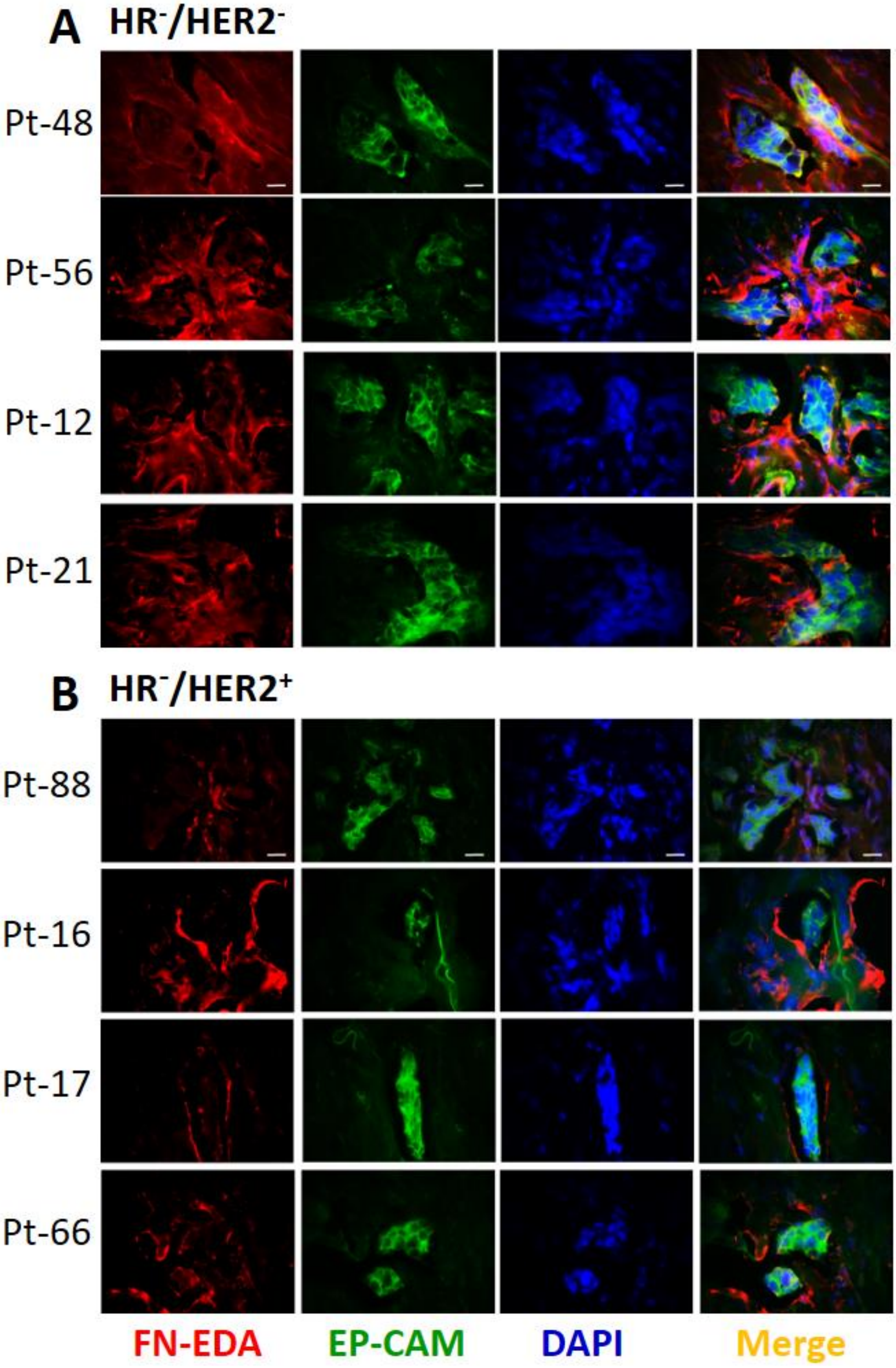
A
HR-/HER2-
Pt-48
Pt-56
Pt-12
Pt-21
B
HR-/HER2+
Pt-88
Pt-16
Pt-17
Pt-66
FN-EDA
EP-CAM
DAPI
Merge

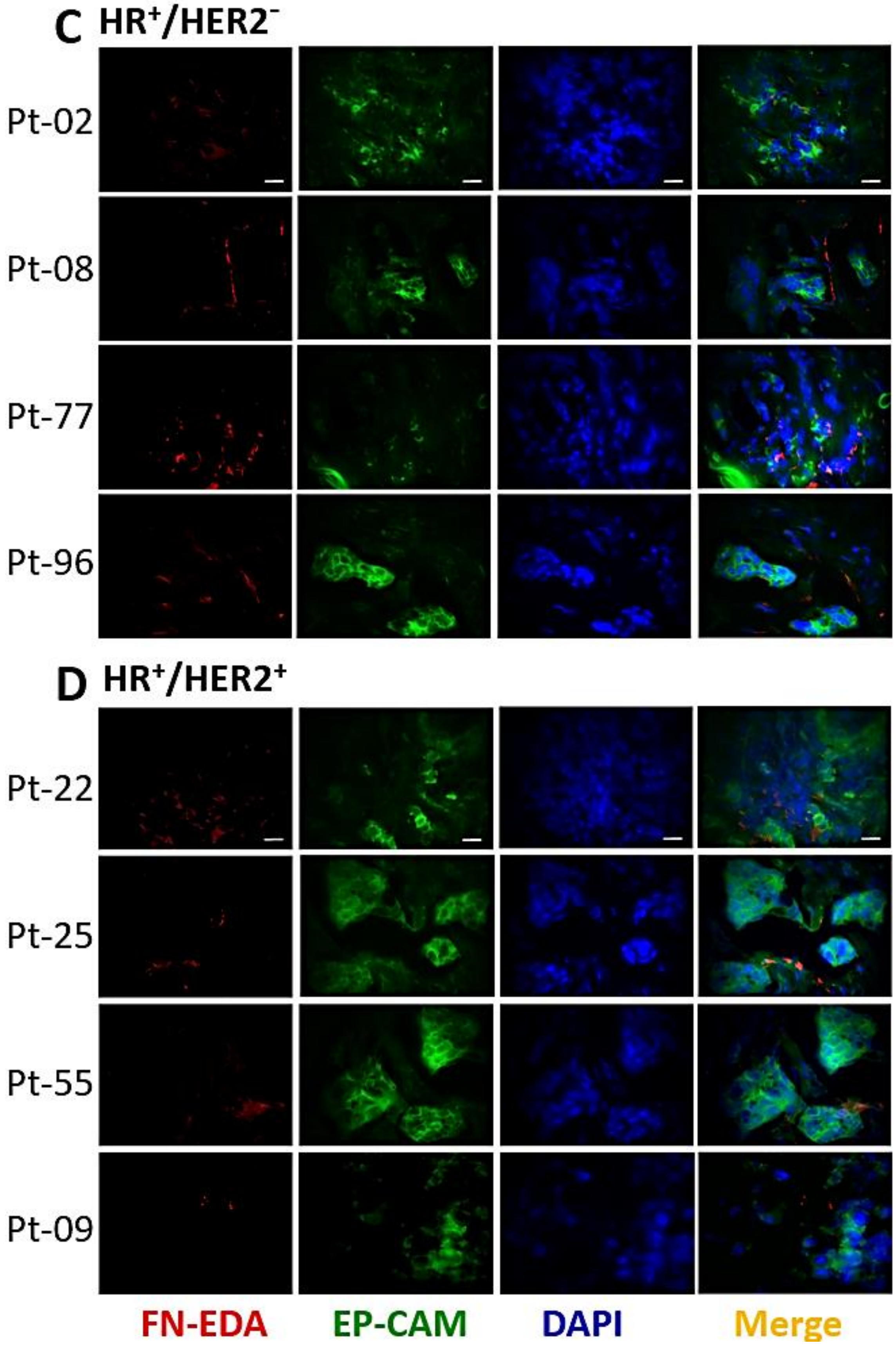

Suppl. Fig. 3. Immunofluorescence analysis of FN-EDA in the breast tumor specimens. Micrographs from four patients who were diagnosed with **(A)** hormone receptor (HR)-negative, HER2-negative (triple negative breast cancer), **(B)** HR-negative, HER2-positive, **(C)** HR-positive, HER2-negative, and **(D)** HR-positive, HER2-positive breast cancer are demonstrated. Epithelial cells are labelled with epithelial cellular adhesion molecule (EpCAM). Please note the co-expression of FN-EDA and EpCAM (appears in yellow color on merged images) in epithelial cells from **(A)** triple negative breast cancer specimens. Scale bar, 10 µm.

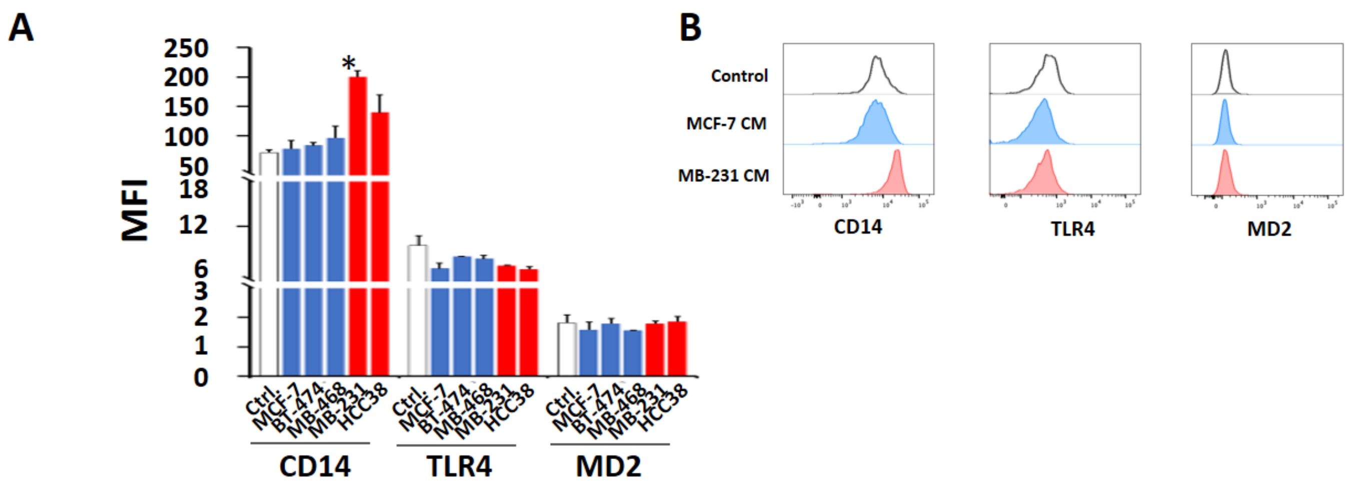

Suppl. Fig. 4. Expression of CD14, TLR4 and MD2 molecules on the monocytes incubated with the conditioned media (CM) from different breast cancer cell lines. **A)** Median fluorescence intensity (MFI) values and **(B)** representative flow cytometry offset plots are shown. (Ctrl., monocytes incubated in the control media; blue bars, CM from low-level fibronectin-expressing cell lines; red bars, CM from high-level fibronectin-expressing cell lines; Mean±SEM, Student's t-test; *, *P* < 0.05)

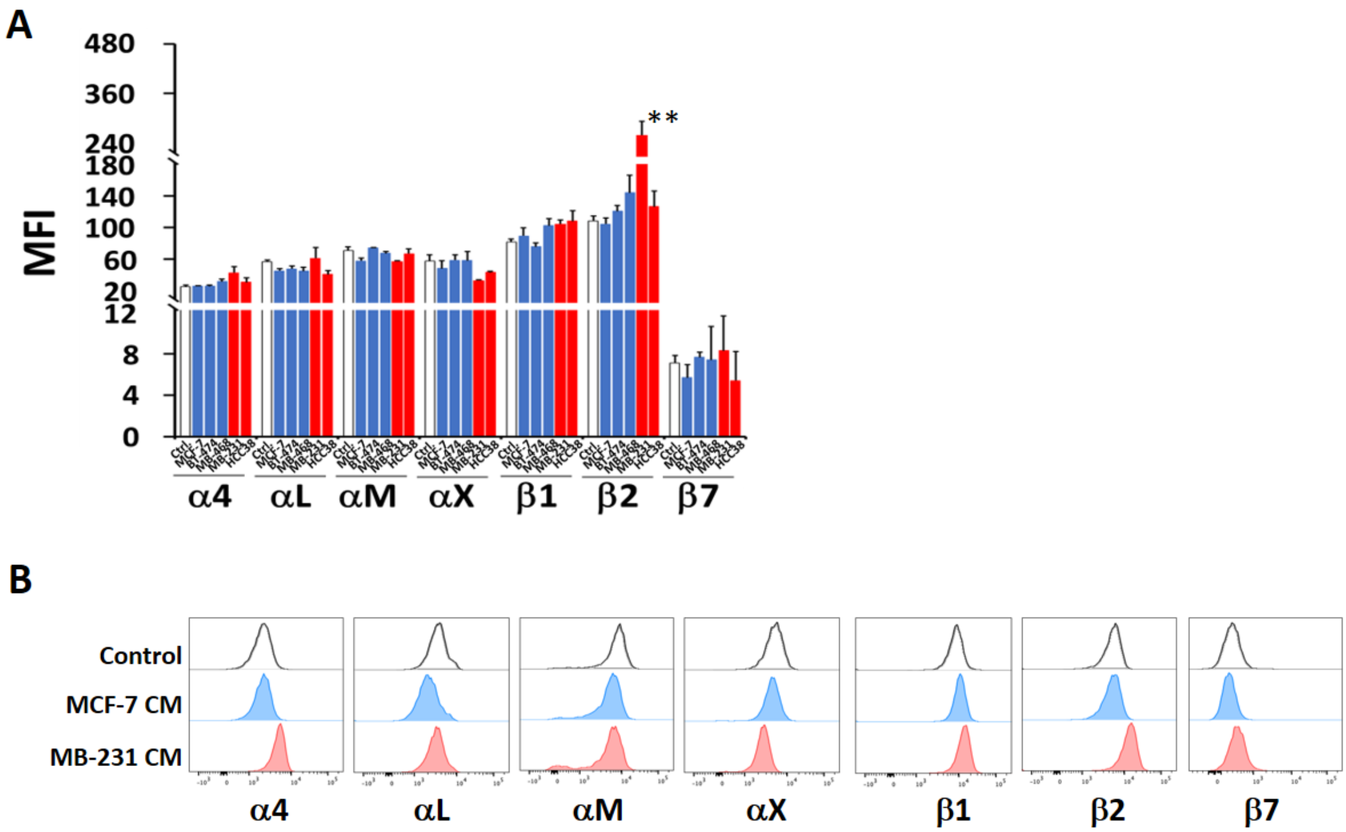

Suppl. Fig. 5. Expression of integrin molecules on the monocytes incubated with the conditioned media (CM) from different breast cancer cell lines. **A)** Median fluorescence intensity (MFI) values and **(B)** representative flow cytometry offset plots are shown. (White bars, monocytes incubated in the control media; blue bars, CM from low-level fibronectin-expressing cell lines; red bars, CM from high-level fibronectin-expressing cell lines; Mean±SEM, Student's t-test; **, $P < 0.01$)

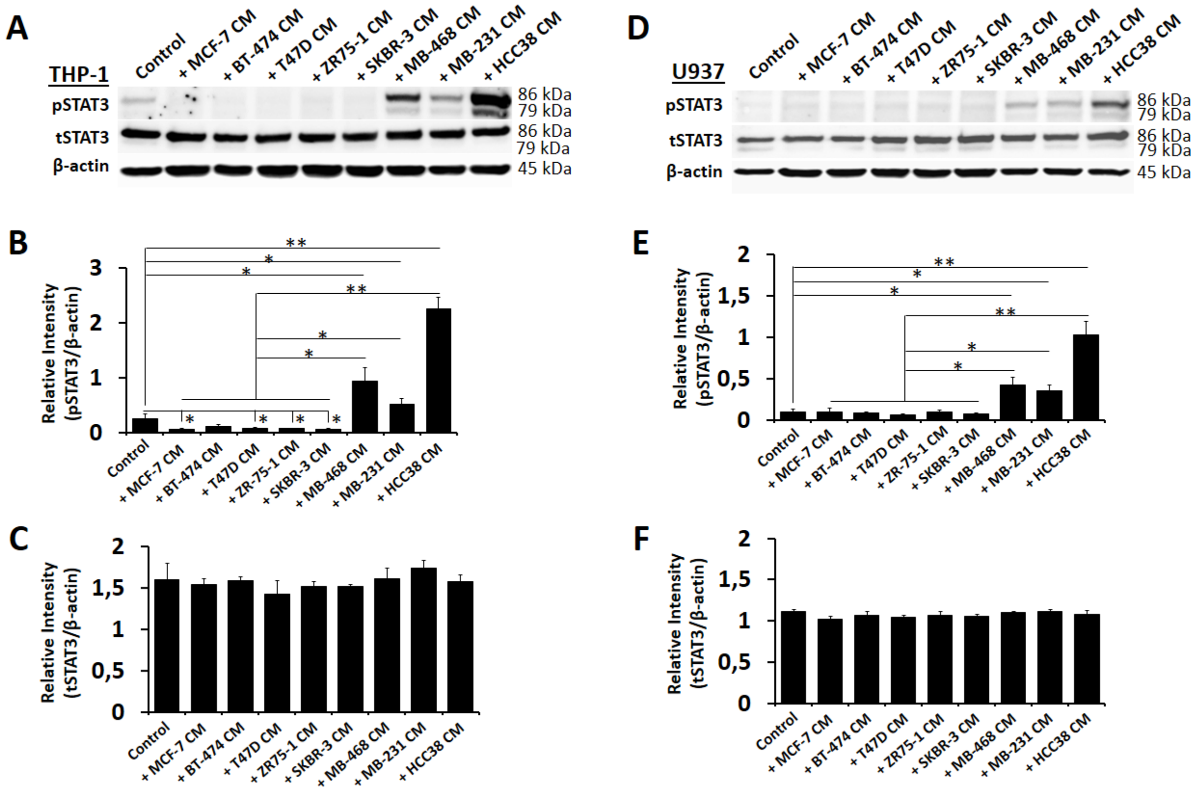

Suppl. Fig. 6. Activation of STAT3 pathway in myeloid **(A-C)** THP-1 and **(D-F)** U937 cell lines following the incubation with conditioned media (CM) from breast cancer cell lines. **A,D)** Representative Western-Blot images and band intensities of **(B,E)** pSTAT3 and **(C,F)** total STAT3 protein normalized according to β-actin are shown. (pSTAT3, phospho-STAT3 (Tyr705); total STAT3, tSTAT3; Mean±SEM, Student's t-test; *, $P < 0.05$ **, $P < 0.01$)

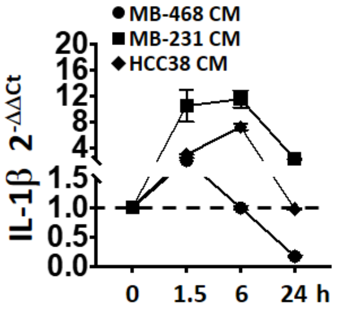

Suppl. Fig. 7. Change in IL-1β gene expression in the THP-1 cells treated with the conditioned media (CM) collected from triple-negative breast cancer cells. The data were normalized to the control THP-1 cells cultured in standard media. The dashed line crossing at $2^{-\Delta\Delta Ct} = 1$ represents an equal expression level between the control and CM-treated cells.

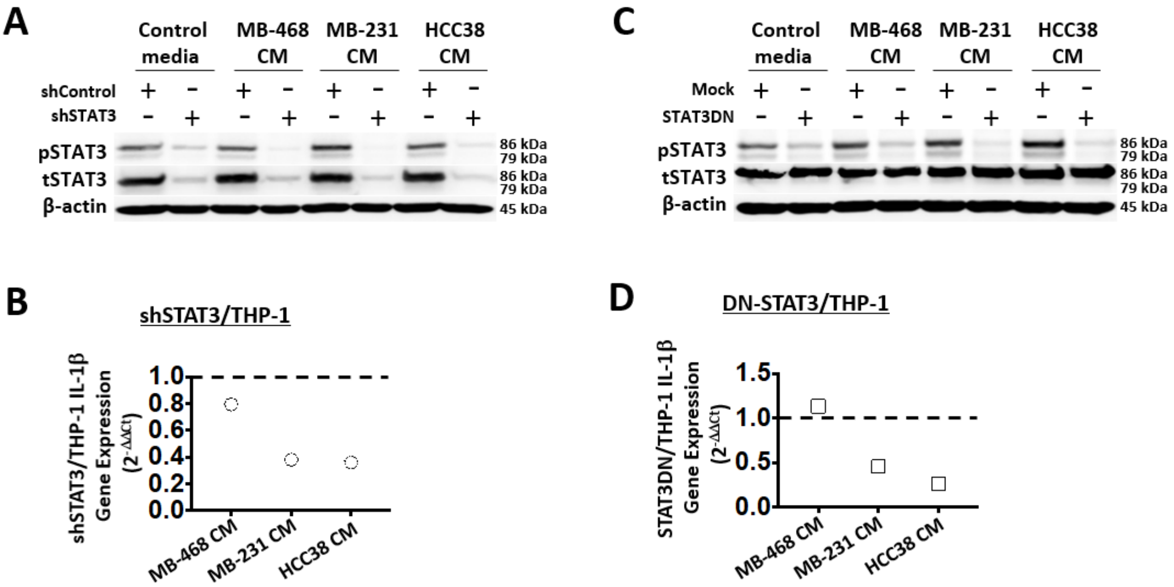

Suppl. Fig. 8. IL-1β gene expression analysis in STAT3 silenced THP-1 cells treated with the breast cancer conditioned media (CM). THP-1 cells were genetically-modified with **(A)** short-hairpin (sh)STAT3 or **(C)** dominant-negative (DN) STAT3 constructs and treated with the CM. pSTAT3 (phospho-STAT3 Tyr705) and tSTAT3 (total STAT3) protein levels were studied by Western-Blot. Change in IL-1β gene expression in **(B)** the shSTAT3-modified and in **(D)** the DN-STAT3-modified THP-1 cells treated with the conditioned media (CM) collected from triple-negative breast cancer cells. The data from the shControl plasmid-modified THP-1 cells and the mock-transfected THP-1 cells were used for the normalization of shSTAT3-modified THP-1 cells and for the DN-STAT3-modified THP-1 cells, respectively. The dashed line crossing at $2^{-\Delta\Delta Ct} = 1$ represents an equal expression level between the control cells and the STAT3-modified cells.
